# Adaptive evolution of benzoxazinoids in wild emmer wheat, *Triticum dicoccoides*, at "Evolution Canyon", Mount Carmel, Israel

**DOI:** 10.1371/journal.pone.0190424

**Published:** 2018-02-06

**Authors:** Yuval Ben-Abu, Avigdor Beiles, Dvir Flom, Eviatar Nevo

**Affiliations:** 1 Projects and Physics Section, Sapir Academic College, D.N. Hof Ashkelon, Israel; 2 Institute of Evolution, University of Haifa, Haifa, Israel; 3 Department of Physics, Ben Gurion University of the Negev, Be'er Sheva, Israel; University of Helsinki, FINLAND

## Abstract

**Background:**

"Evolution Canyon" (ECI) at Lower Nahal Oren, Mount Carmel, Israel, is an optimal natural microscale model for unraveling evolution-in-action, highlighting the evolutionary processes of biodiversity evolution, adaptation, and incipient sympatric speciation. A major model organism in ECI is the tetraploid wild emmer wheat, *Triticum dicoccoides* (TD), the progenitor of cultivated emmer and durum wheat. TD displays dramatic interslope adaptive evolutionary divergence on the tropical, savannoid-hot and dry south-facing, "African" slope (AS), and on the temperate, forested, cool and humid, north-facing, "European" slope (ES), separated on average by 250 m. From the perspective of chemical evolution and metabolomics, it is important to unravel interslope divergence in biologically relevant secondary metabolites between the abutting slope populations. Here, in TD we examined hydroxamic acid (Hx), which is a family of secondary cereal metabolites, and plays a major role in defending the plant against fungi, insects and weeds.

**Results:**

Our examination revealed that higher concentrations of DIBOA and DIMBOA were found in seedlings growing in the same greenhouse from seeds collected from the cool and humid forested ES, whereas the seedlings of seeds collected from the savannoid AS (both in root and shoot tissues), showed no DIMBOA. Remarkably, only DIBOA appears in both shoots and roots of the AS seedlings. It rises to a peak and then decreases in both organs and in seedlings from both slopes. The DIMBOA, which appears only in the ES seedlings, rises to a peak and decreases in the shoot, but increased and remained in a plateau in the root, till the end of the experiment.

**Conculsions/Significance:**

The results suggest stronger genetic resistance of defense compounds DIBOA and DIMBOA against biotic stresses (fungi and other pathogens) by ES seedlings. However, AS seedlings responded earlier but were to the same biotic stresses. The genetic difference found in AS seedlings was caused by the main adaptive selection in AS, which was against climatic, abiotic stresses, and was weaker, or not at all, against biotic stresses. The distinct genetic interslope differences appear important and is very significant and are elaborated in the discussion.

## Introduction

**Fig 1:**
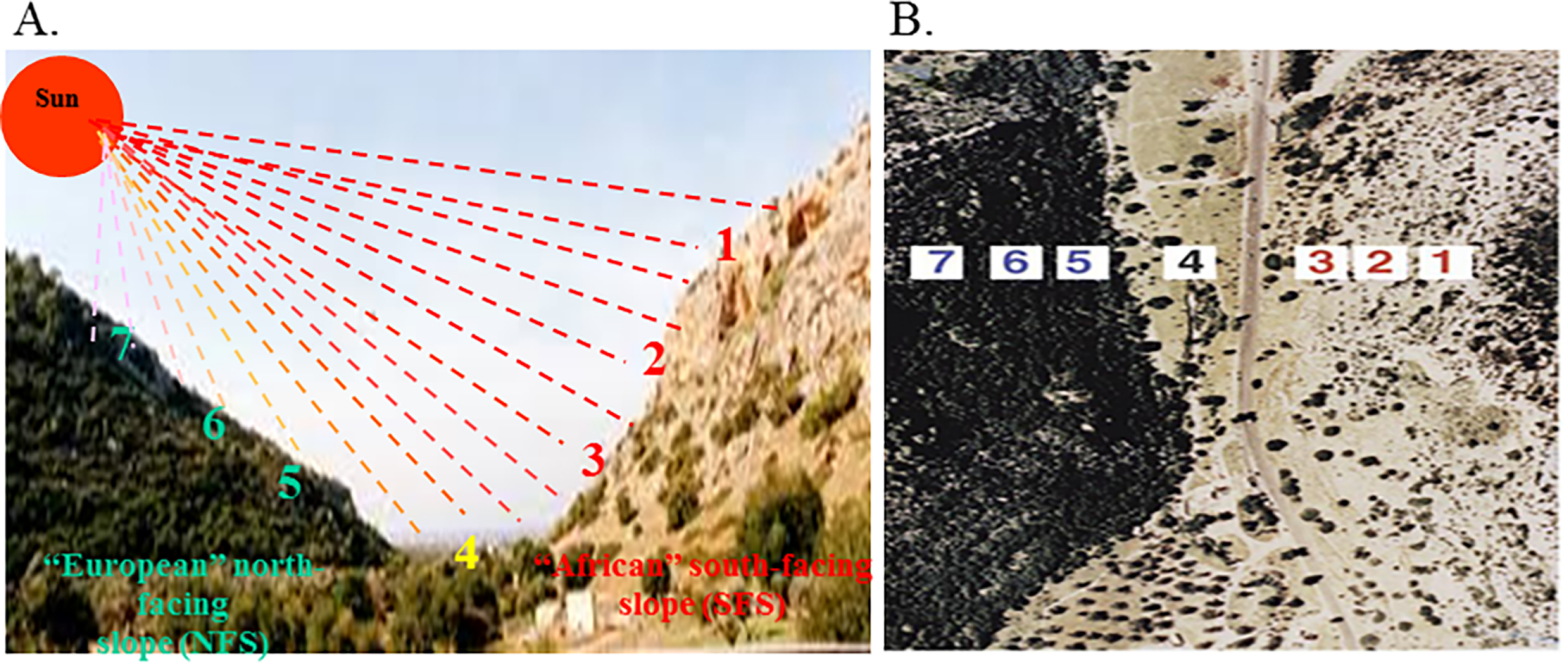
“Evolution Canyon” I (ECI) at Lower Nahal Oren, Mount Carmel, Israel. **(A)** Cross section of “Evolution Canyon” showing the savannoid "African" slope (AS) = south-facing slope (SFS), and the opposite shady, forested "European" slope (ES) = north-facing slope (NFS). (B) Air view of ECI with the forested NFS = ES and the savanoid SFS = AS, with the 7 experimental stations where the *T*. *dicoccoides* was sampled, indicated by the numbers 1–7 (from ref. 7, private picture of Prof. E. Nevo).

The "Evolution Canyon" (ECI) microsite model is the focus of a long-term research program that began in the Lower Nahal Oren, Mount Carmel, Israel in 1990 and was extended to three additional ECs: Upper Galilee (ECII) [1–3], southern Negev desert [2, 4–5] (EC III), and the Golan (ECIV) [4–6]. The "Evolution Canyon" (EC) microscale model reveals evolution in action at a microsite involving biodiversity evolution, adaptation, and incipient sympatric ecological speciation across life from viruses and bacteria through fungi, plants and animals [4,7,8]. The AS and ES exhibit drought and shade stresses, respectively. The EC is an ideal microscale natural laboratory for the study of microscale evolution in action via a model that highlights the basic evolutionary processes of adaptation and speciation. *Triticum dicoccoides*, TD, displays dramatic interslope adaptive evolutionary divergence on the tropical, savannoid-hot and dry south-facing, "African" slope (AS), and on the temperate, forested, cool and humid, north-facing, "European" slope (ES), separated on average by 250 m [9–12] (Fig 1A and 1B). Terrestrial species are more abundant on the harsher tropical AS, while aquatic species are more abundant on the ES [1, 13,14]. The major environmental stresses on the AS are solar radiation, temperature and drought, all of which are higher on the AS than on the ES, and associated with higher genetic variation, on average, on the AS than on the ES [3, 7, 15, 16]. This microscale pattern of genetic variation positively associating with environmental stress is true locally, such as in ECI, regionally, across Israel, and globally [16]. Ecological stress at a microscale can generally generate regional or global-scale effects of phonemics, genomics and proteomics, reinforcing homeostasis and fitness, thereby suggesting continuity between micro- and macroevolution [1, 7, 16].

The defense compounds are present in wheat and maize, as well as in a wide range of wild Gramineae. In wheat and maize, the hydroxamic acid derivative 2,4-dihydroxy-7-methoxy-1,4-benzoxanin-3-one (DIMBOA) [17–21] plays a major role in defending the plant against fungi [21, 22], insects [19, 23], and weeds [24]. The growth of aphids and their populations on wheat and maize plants correlate negatively with plant DIMBOA levels [22], as the compound is toxic and alters the feeding behavior of aphids, both on the wheat plants *in vivo* and in the context of an artificial diet [23].

**Fig 2:**
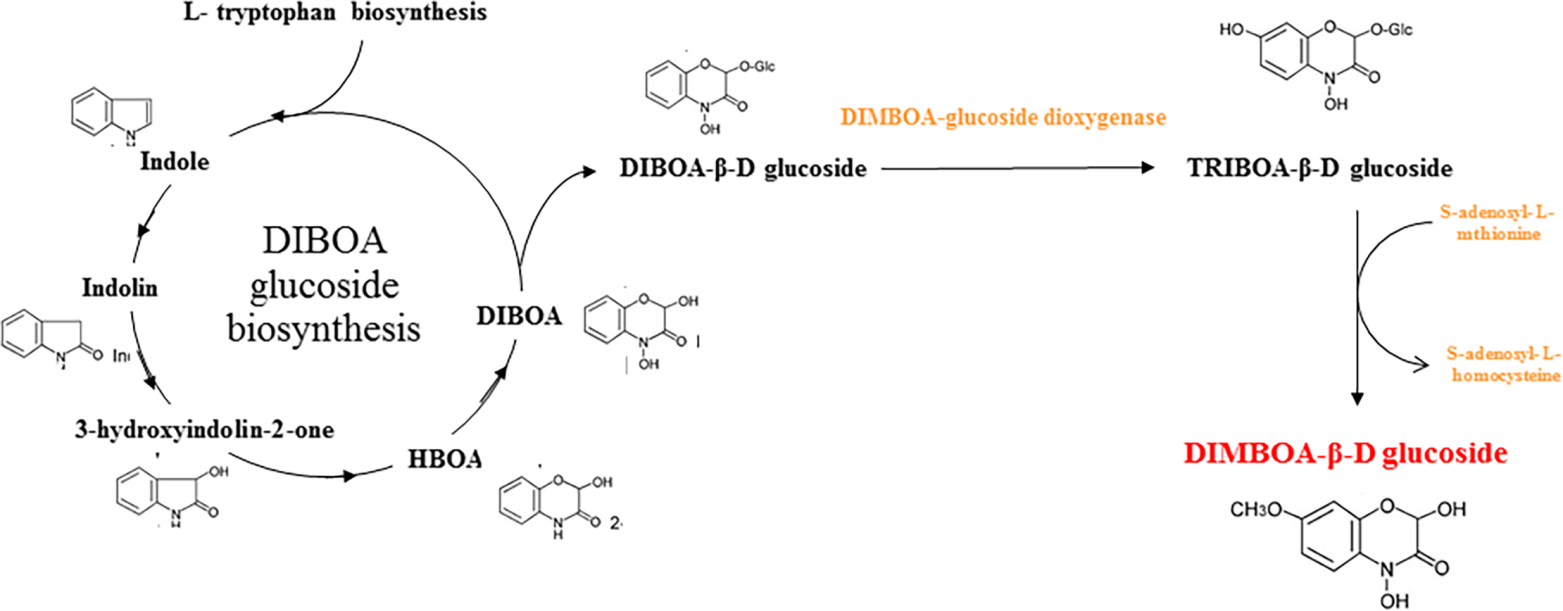
Schematic representation of the Bx biosynthetic pathway showing enzymatic reactions catalysed by the gene products of *TaBx1*-*TaBx5* (in the plastids and microsomes) in hexaploid wheat [17].

From the perspective of chemical evolution, the main question that needs elaboration at ECI concerns differences in biologically relevant interslope secondary metabolites and chemical substances. Here, we examined interslope divergence of hydroxamic acid (Hx) in two wild emmer wheats, *Triticum dicoccoides* (TD) populations growing on the opposite abutting, ecologically sharply divergent slopes of ECI. The hydroxamic acids are a family of secondary metabolites of cereals discovered over four decades ago in a relative of rye (Fig 2, adapted from ref. [17]).

Several studies have shown that benzoxazinoids and their metabolites can inhibit weed germination and root growth. Specifically, DIMBOA and its metabolite 6-methoxybenzoxazolin-2-one (MBOA) inhibit seed germination of the weed *Avena fatua*, with DIMBOA being more potent than MBOA [17, 24]. Similar findings were reported with respect to the activity of 2,4- dihydroxy-1,4-benzoxazin-3-one (DIBOA) and its metabolites (Fig 2) [17]. Here we studied the benzoxazinoid compounds in seedlings from two interslope populations of wild emmer wheat, *Triticum dicoccoides*, at ECI, in Mount Carmel, Israel. We demonstrated dramatic quantitative and qualitative divergence of the production of benzoxazinoid compounds in response to the same trigger in the same greenhouse between the seedlings collected from the abutting slopes at ECI: AS versus ES. This remarkable adaptive pattern relates to the humid and cooler microclimate on the ES, where fungi flourish and, we assume, that adaptively higher DIMBOA and DIBOA concentrations are generated by the ES seedlings to resist their pathogens and biotic diseases. It is important to note that most defense compounds are produced only when there is an attack or a stress, i.e., a trigger, to start the production. Thus, the observed shoot–root difference, in the same seedling, with the same genetic background described in the results is caused by different pathogen attacks from the soil or from the air.

## Materials and methods

The seeds source: The ECI consists of two abutting slopes separated on average by 250m. The tropical, hot and dry savannoid "African" south-facing slope (AS = SFS) abuts with the temperate, cool and humid, forested "European" north-facing slope (ES = NFS). The AS receives 200–800% higher solar radiation than the ES [3]. The seeds of wild emmer wheat have been collected from amid-slope station (population) #2 on the AS = SFS and the highest station #7 on the ES = NFS, and tested in a greenhouse (see later).

### Plant materials

We collected 10 accessions (genotypes), 5 in each station (population) on each slope: station #2 on the AS and station #7 on the ES of wild emmer wheat, *Triticum dicoccoides*, from ECI, Mount Carmel, Israel (see the contrasting populations in Fig 1.) On average, we grew 50.4 seeds from each of the 10 genotypes, totaling 504 seeds, generating 504 seedlings. Each of the measurements of the concentration of the defense compounds (the procedure will be described later) has been conducted on 3 roots or 3 shoots from 3 seedlings of the same genotype (168 mixtures consisting of 84 mixtures from each slope). Each of the 84 mixtures have been subdivided into 7 repetitions of 12-time measurements, from 24 hours after seeding up to 144 hours after seeding. The time points were 24, 30, 36, 48, 60, 72, 84, 96, 108, 120, 132, and 144. The space between each measurement was 12 hours, except in the first 3 measurements that were conducted every 6 hours. If the four first replicates were all zeros, we terminated measuring and assumed that the last 3 measurements were also zeros. In the AS seedlings, all DIMBOA were zeros and all DIBOA appeared. By contrast, in ES seedlings, the first two measurements were zeros in both DIBOA and DIMBOA; hence, the final slope measurements were different: 78 on the ES and 84 on the AS. All entries of Table 1 consist of three seedlings of the same genotype grown in three different sites in the greenhouse in order to achieve more reliable measurements.

**Table 1:** Variation in the amounts of Bx (DIBOA and DIMBOA) in *T*. *dicoccoides* seedlings (nmol/mg FW) grown in Sapir greenhouse from seeds collected on the opposing slopes, AS and ES, of “Evolution Canyon”. The measurements were separately done in shoots and roots of each seedling. Each average, standard error and median appearing in the table were calculated from 7 replications of the measuring at the same time. The replications appear in a separate column in the table. When the first 4 replications had 0 Bx, we terminated this analysis and assumed that the 3 unmeasured replications are also zero. Mean +/- Standard error values of the 7 replications measured each time, and Median values of the replications. Abbreviations: AS = African slope, ES = European slope, Bx = Benzoxazinoid. Glc = Glucosidase, Fw = Fresh Weight, nmol. = Nano mole, Rep. (or R.) = Replicates. Note: Each replication in the table is the result of measuring a mixture of 3 seedlings from the same genotype in order to receive more reliable amounts.

	AS seedlings								ES seedlings							
Hours after seeding	Shoot (nmol/mg FW)				Root (nmol/mg FW)				Shoot (nmol/mg FW)				Root (nmol/mg FW)			
	Rep.	DIMBOA-Glc Mean ± SE (Median)	Rep.	DIBOA-Glc Mean ± SE (Median)	Rep.	DIMBOA-Glc Mean ± SE (Median)	Rep.	DIBOA-Glc Mean ± SE (Median)	Rep.	DIMBOA-Glc Mean ± SE (Median)	Rep.	DIBOA-Glc Mean ± SE (Median)	Rep.	DIMBOA-Glc Mean ± SE (Median)	Rep.	DIBOA-Glc Mean ± SE (Median)
24	0 0 0 0	0±0 (0)	0.1 0.10.2 0 0.2 0	0.1±0.03 (0.1)	0 0 0 0	0±0 (0)	0.33 0.28 0.37 0.3 0.25 0.22 0.35	0.1±0.03 (0.1)	0 0 0 0	0±0 (0)	0 0 0 0	0±0 (0)	0 0 0 0	0±0 (0)	0 0 0 0	0±0 (0)
30	0 0 0 0	0±0 (0)	0.32 0.31 0.35 0.32 0.33 0.37 0.45	0.35±0.018 (0.33)	0 0 0 0	0±0 (0)	0.42 0.48 0.6 0.52 0.5 0.55 0.71	0.54±0.03 (0.52)	0 0 0 0	0±0 (0)	0 0 0 0	0±0 (0)	0 0 0 0	0±0 (0)	0 0 0 0	0±0 (0)
36	0 0 0 0	0±0 (0)	0.5 0.7 1 0.4 0.5 0.3 0.3	0.528±0.09 (0.5)	0 0 0 0	0±0 (0)	1.2 0.8 0.8 0.7 1 1.2 1.2	0.98±0.08 (1)	0.25 0 0.3 0.9 0 0.9 0.5	0.4±0.148 (1)	0 0 0 0	0±0 (0)	0.2 0.3 0.2 0.4 0 0.5 0.3	0.27±0.06 (0.3)	1.5 1.2 1 0.2 0.4 0.6 0.9	0.82±0.12 (0.9)
48	0 0 0 0	0±0 (0)	2.7 3 2.5 2.4 2.1 3 1.8	2.5±0.16 (2.5)	0 0 0 0	0±0 (0)	6.5 7 5.4 6 6.1 5.9 6.5	6.2±0.195 (6.1)	3 4 3.5 2.6 3 3 2.6	3±0.183 (3)	0 0 0 0	0±0 (0)	3 2 2 4 1.5 3 2	2.5±0.32 (2)	6 6 3 4 5 5 7.4	5.2±0.43 (5)
60	0 0 0 0	0±0 (0)	1.7 2.1 1.9 1.1 1.5 1.4 0.8	1.5±0.17 (1.5)	0 0 0 0	0±0 (0)	6.6 7 7 75 7.3 5.54 7	6.5±0.329 (7)	10 9 9 9 7 8 11	9±0.16 (9)	1.7 2 1 1 1.5 1.3 2	1.5±0.16 (1.5)	3 3 3 4 4 4 7	4±0.32 (4)	10 10 7 8 9 8 11	9±0.56 (0)
72	0 0 0 0	0±0 (0)	0.5 0.4 0.3 0.5 0.4 0.5 0.9	0.5±0.07 (0.5)	0 0 0 0	0±0 (0)	5 4 6 4 4.3 5 3.9	4.6±0.291 (4.3)	13 13 12 11 15 10 17	13±0.89 (13)	2.5 2 3 2.5 2 3.5 3.4	2.7±0.232 (2.5)	5 4 4 3 5 4 3	4±0.406 (4)	12 15 13 13 9 14 15	13±0.507 (13)
84	0 0 0 0	0±0 (0)	0.4 0.4 0.8 0.6 0.5 0.5	0.6±0.804 (0.5)	0 0 0 0	0±0 (0)	3 3 5 4 3 4 6	4±0.435 (4)	12.5 12 11 11 13 12 12.5	12±0.386 (15)	3 3 3 4 5 4 6	4±0.435 (4)	5 5 3 6 6 2.5 8.2	5.1±0.786 (5)	10 12 14 12 11 14 9.6	11.8±0.665 (12)
96	0 0 0 0	0±0 (0)	0.5 0.4 0.3 0.4 0.5 0.4 0.3	0.4±0.03 (0)	0 0 0 0	0±0 (0)	5 5 4 3 2.5 5 4.9	4.2±0.401 (4.9)	8 7 11 9 9 9 10	9±0.187 (9)	12 10 7 9 10 10 12	10±0.654 (10)	4 4 4 3 6 6 8	5±0.6 (4)	8 8 6 5 7 7 8	7±0.436 (7)
108	0 0 0 0	0±0 (0)	0.3 0.3 0.3 0.2 0.2 0.4 0.4	0.3±0.03 (0.3)	0 0 0 0	0±0 (0)	2 3 4 3 3 3 3	3±0.218 (3)	7 6 6 6 5 7 5	6±0.383 (6)	9 9 9 7 10 8 11	9±0.487 (9)	6 6.7 6 7 5.1 2 4.3	5.2±0.65 (6)	7 7 8 6 6 5 6.5	6.5±0.37 (6.5)
120	0 0 0 0	0±0 (0)	0.4 0.4 0.5 0.2 0.2 0.3 0.8	0.4±0.07 (0.4)	0 0 0 0	0±0 (0)	3 2 2 4 2 3 2.9	2.7±0.09 (2.9)	3 2 2 2 3 1.5 3.65	2.45±0.29 (2)	8 8 8 6 5 9 12	8±0.845 (8)	6 5.5 7 4 4 4 6.6	5.3±0.492 (5.5)	5 5 4 3 7 2.5 3.6	3.4±0.06 (4)
132	0 0 0 0	0±0 (0)	0.4 0.5 0.1 0.3 0.2 0.2 0.4	0.3±0.03 (0.3)	0 0 0 0	0±0 (0)	3 3 2 2 2 3 2.5	2.5±0.108 (2.5)	0.5 0.5 0.5 0.4 0.5 0.4 0.7	0.5±0.01 (0.1)	6 5 9 7 7 7 8	7±0.74 (7)	6 6 5 4 4 6 8	5.7±0.62 (6)	4 2 2 2 2.5 2 3	2.5±0.32 (2)
144	0 0 0 0	0±0 (0)	0.4 0.4 0.4 0.2 0.2 0.3 0.3	0.3±0.03 (0)	0 0 0 0	0±0 (0)	3 3 3 2 2 2 1.8	2.4±0.213 (2)	0.2 0 0 0.2 0.1 0.1 0.1	0.1±0.08 (0.1)	5 5 5 4 7 6 3	5±0.48 (5)	5 5 4 4 7 5 6.4	5.2±0.404 (5)	3 3 2 1.5 1 2 2.2	2.1±0.245 (2)

The conducted field studies did not involve endangered or protected species, and permission was received from the “Israel Nature Reserve Authority", the body responsible for this site. Shoots and roots excised from the seedlings were frozen in liquid nitrogen and were grounded separately into fine powders by mortar and pestle.

### Benzoxazinoid extraction and HPLC analysis

Samples of *T*. *dicoccoides* were taken from AS and ES slopes and were grown in the greenhouse. The wheat seeds collected from the AS and ES were placed randomly at different places of the greenhouse. Benzoxazinoids were extracted by adding HOAc:MeOH (2:98, vol/vol). The extract was passed through a filter (Millex, Millipore) and subjected to HPLC using a Wakosil II 5C18 HG (4.6 × 150 mm) column. Elution was performed with 22% (vol/vol) MeOH in 0.1% (vol/vol) HOAc at a flow rate of 0.8 ml/min, at a temperature of 40°C. Eluted proteins were detected at 280 nm [25–27]. We also used MATLAB 8.0 and Statistics Toolbox 8.1 software to illustrate the frequency distribution of the DIMBOA and DIBOA amount.

The MATLAB script: The MatLab script was added as an appendix [17].

### Statistical analysis

We used 2 nonparametric tests because we have no proof and no indication that normality is shown in the measured data. In each measurement we averaged 7 independent results of new seedlings and this sample was too small for normal approximation. We used sign test in all cases where we did pairwise comparisons. We used the Mann Whitney test in all cases comparing two averages. Our weaker tests mostly yielded low p’s, i.e., high significance.

## Results

**Fig 3:**
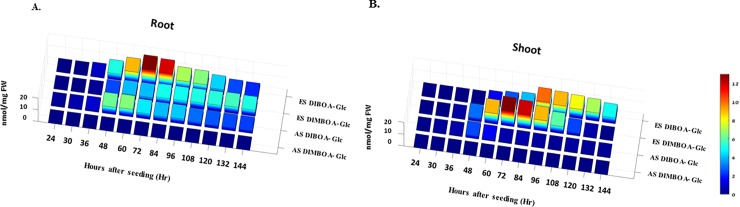
Diagram showing changes of the amounts (nmol/mg FW) of Benzoxazinoids (Bx) in seedlings of emmer wheat, *Triticum dicoccoides*. Seeds were collected in the field at Evolution Canyon I, Lower Nahal Oren, Mount Carmel, Israel. Seeds were germinated in a greenhouse (i.e., in a common garden experiment). Changes in DIBOA and DIMBOA concentration**s** were measured mostly in each 12-hour starting from 24 hours till 144 hours after seeding, showing the concentration of DIBOA and DIMBOA (A) in roots (B) in shoots. Each value is an average of 7 mixtures, each of 3 seedlings, from the same genotype. Abbreviations: AS = African slope, and ES = European slope at Evolution Canyon (ECI). Glc-glucoside, FW- fresh weight.

Our examination revealed that, generally, higher concentrations of DIBOA and/or DIMBOA were found in both roots and shoots in seedlings of wild emmer wheat, *Triticum dicoccoides*, (TD), growing in a greenhouse from seeds collected from plants growing on the temperate cool and humid ES, than in seedling growing from seeds collected on the tropical, hot and dry AS. We use the term “generally”, because it sums up 2 antagonistic results: Only the AS seedlings produced DIBOA during the 2 first measurements, thus, they responded earlier than the ES seedlings. In those first 2 measurements, the AS>ES. But, after two more measurements, in the last 8 measurements, the ES seedlings produced significantly more defense compounds (DIBOA + DIMBOA) than the AS seedlings, i.e., ES>AS: (Table 1 and Fig 3). The ES seedlings generally generated more of both defense compounds. If roots (or shoots) in those 8 measurements are tested by sign test, then the difference between the slopes is significant, p< 0.01. By contrast, generally, the opposite, i.e., mostly lower concentrations of DIBOA and no DIMBOA at all, were found in seedlings, also growing in the same greenhouse, from seeds collected on the abutting tropical hot and dry AS (Table 1 and Fig 3A and 3B).

A particularly detailed analysis (Table 1 and Fig 3A and 3B) indicates that DIMBOA in both roots and shoots was zero in all of the AS seedlings and was found only in the ES seedlings. That difference is significant (p<0.0005, tested by sign test). On the other hand, in the ES seedling, DIMBOA in shoots rose significantly (see Table 1) to the highest peak (13 nmol/mg FW) and then decreased to almost zero (0.1 nmol/mg FW), whereas in roots it rose significantly (see Table 1) to an intermediate level and stayed near that intermediate level till the end of our experiment, thus creating a plateau between 5 to 5.7 nmol/mg FW (Table 1, Fig 3A). DIBOA in shoots appeared in the AS seedlings in low concentrations and started decreasing from the lowest peak (2.5 nmol/mg FW) at 48 h after seeding to almost zero (0.3 nmol/mg FW). DIBOA in roots appeared in the AS seedlings, peaking at 6.5 nmol/mg FW at 60 h, then decreasing to 2.4 nmol/mg FW. In the ES seedlings DIBOA in shoots peaked at 10 nmol/mg FW and then declined to 5 nmol/mg FW; in roots, DIBOA peaked at 13 nmol/mg FW (the same highest peak as the DIMBOA in shoots) and then decreased to 2.1 nmol/mg FW, which was less than what was found in roots of the AS seedlings.

In the ES seedlings, starting from the 3^rd^ measurement, both compounds appeared in roots, but only DIMBOA appeared in shoots. The DIBOA accumulation in shoots started only from 60 hours on after seeding (the 5^th^ measurement, see Table 1). In contrast, in AS seedlings DIBOA appeared from the first measurement 24 h after seeding. We hypothesize that the greenhouse environment is as wet as in the ES but not as dry as the AS. This is probably why the ES seedlings respond later and weaker at the beginning than the AS seedlings. Moreover, although the DIBOA root concentration in ES seedlings was higher only during the first 72 hours of the experiment, the amount was much higher compared to the AS seedlings (see all detailed measurements in Table 1, and the global pattern in Fig 3).

## Discussion

### Overall results and their explanation

What caused the differences between the DIMBOA and DIBOA concentrations in the seedlings from the AS and ES taken from two sharply micro-climatically contrasting slopes at ECI but grown in the same greenhouse (i.e., common garden) and between the two organs of the same seedling? The interslope variation at ECI in the collected seeds in DIMBOA and DIBOA may be explained by the genetic and epigenetic adaptations to the differences of the biotic stresses on the ES and the abiotic stresses on the AS in the abutting ECI slopes caused by the different microclimates (temperate versus tropical). The rocks, as well as the soil type on both slopes are limestone and terra rossa [26], but the interslope microclimate variation [3] leads to a high humidity divergence both in soil and air of the ES, leading to higher humidity and organic material on the ES [25, 26]. The result is biotic adaptation to a temperate humid and cool microclimate on the ES, which is rich in biotic stresses, i.e., pathogens, to the contrasting abiotic, hot, and dry AS. The ES soil is rich in bacteria and fungi, whereas the air abounds with spores of powdery mildew and rusts. The difference between soil and air pathogens, both in the EC and in the greenhouse, may also explain the increases and decreases of DIMBOA in shoots and its later constancy in roots of the ES seedlings. The interslope sharp microclimatic divergence is caused by the sun shining up to 800% more solar radiation on AS, thereby causing higher temperatures and drought on the SFS = AS (Fig 1A and 1B) [3, 13,14].

To highlight the above explanatory model, we can say that the ES soil constitutes a hydrated environment that contains high quantities of bacteria and fungi. The latter may be one of the main reasons for the greater quantities of ES root DIMBOA as compared to the AS (Table 1 and Fig 3). This hypothesis has been previously confirmed for other cereals such as maize, rice, and especially wheat. An earlier assessment of DIMBOA amounts in cereals grown in a desert area (similar to the savannoid AS) reported the presence of only very low levels of this compound [28]. These previously reported results could explain why there was no DIMBOA detected in seedlings collected from the AS. Moreover, experiments conducted on cereals showed that DIMBOA levels in moist and hydrated soil and humid air, such as on the ES at ECI were higher than those detected in a dry habitat [29]. As such, since the humid and cool ES represents a temperate and forested slope, only seedlings collected at the ES had high amounts of DIMBOA but none were found in seedlings from AS seeds, possibly as the result of many more pathogenic organisms such as fungi [30–32]. The abundance of fungi, such as powdery mildew and rusts on the ES due to cool and humid environment, would cause plants in this temperate microclimate [32–35] to develop the genetic ability to produce more defense compounds like DIBOA and DIMBOA as a protective adaptation against environmental biotic threats [32].

### The difference between shoots and roots

One of the viewpoints that arise from the differences between the DIMBOA and DIBOA concentration in roots and shoots, which are presented in Fig 3, could be explained by Fig 2.

In the roots of maize plants DIMBOA concentrations were lower than those of DIBOA [33, 36–39]. Since the amount of DIMBOA is a function of the amount of DIBOA and the levels of each are relative to the other, we speculate that DIMBOA synthesis occurs in response to a trigger. When the plant senses ‘danger’ in its environment, its tissues are stimulated to produce DIMBOA from DIBOA [36–38]. This production continues until the levels of DIMBOA and DIBOA are equal, although in some cases the amount of DIBOA exceeds that of DIMBOA [37]. In 50% of the measurements taken in our experiment with shoots and in 30% of the cases at the end of the measurements in roots, DIMBOA exceeds the amount of DIBOA (Table 1, ES seedlings). In the first appearance time of the benzoid experiment, ES seedlings of shoots had only DIMBOA without any evidence of DIBOA. It seems that in wild wheat the amount of the defense compounds depends on the trigger and not on the DIBOA concentration, which is in contrast to maize and similar to rye. Another interesting fact is that in the ES seedling, the DIBOA accumulation in shoots started only from 60 hours after seeding. We hypothesize that the greenhouse environment is as wet as in the ES which is in contrast to drought conditions of the AS. This may explain the later response of the ES seedlings. Moreover, although the DIBOA root concentration of ES seedlings was high during the first 60 hours, the amount was higher only compared to the concentration of AS seedlings.

### Evolution Canyon (ECI) as a host-pathogen model

What causes DIMBOA and DIBOA accumulations in our *T*. *dicoccoides* plant at ECI? Many studies show that fungi are one of the main causes for DIMBOA and DIBOA accumulations [30–35]. Remarkably, Yin et al. [32} did research on wild emmer wheat at the Evolution Canyon microsite at Mount Carmel, Israel, and revealed that all plants grown on the AS (dry and hot slope) did not develop resistance and were susceptible to powdery mildew, a common plant disease, whereas plants derived from the ES (cool and humid slope) developed genetic disease resistance against powdery mildew. This research [32] showed that a local ecological stress on the ES caused by microclimatic interslope divergence [1] can generate adaptive evolutionary genome responses at *micro* scale reinforcing fitness and suggesting continuity between micro and macro–evolution on a regional or global scale. Additionally, according to this research we can learn and better understand chemical evolution. Importantly, we conclude that powdery mildew research [32] can highlight our current results. The ES at ECI appears to be a cradle for the evolution of disease resistance. Both, Yin et al. [32] and the current study indicate that a microscale model like Evolution Canyon can be a fitting host-pathogen model in the evolution of resistance against pathogens and is also a model of biodiversity evolution, adaptation and incipient sympatric ecological speciation.

### The Evolution Canyon as a multiple evolutionary model

By analyzing the genetic diversity in and between populations and species from bacteria to mammals of the AS and ES at ECI, the following general conclusions have been drawn [1–15, 32]. First, microclimatic selection appears to be the major evolutionary interslope, fast-acting, and diverging force affecting the adaptation of genotypes and phenotypes. Second, selection overrides migration and genetic drift. Third, genetic variation was higher in 16 model organisms on the more stressful AS than on the milder ES, which correlates with the high abiotic stress caused on this African biome [1]. Fourth, incipient sympatric ecological adaptive speciation occurs in model organisms examined at ECI [8]. The EC evolutionary model could be a useful microscale model to monitor global warming. Finally, The ECI, can be an exciting host-pathogen model as shown for wild emmer wheat *Triticum dicoccoides* and the powdery mildew fungal pathogen [32] in wild barley (*Hordeum spontaneum*) and rust (ongoing research). Last but not least, the hydroxamic acid family of DIBOA and DIMBOA which are secondary cereal metabolites, highlight their potential importance at the Evolution Canyon model in defending plants against pathogens.

## Conclusion and prospects

This study showed that the genetics of benzoxazinoid production in two contrasting populations at the AS and ES of wild emmer wheat *T*. *dicoccoide*, differ largely and significantly when tested in the same greenhouse, i.e., the same environment. The ES, largely in both in roots and shoots of seedlings, responded much stronger with both DIMBOA and DIBOA, while the AS seedling responded earlier but was weaker and only with DIBOA. The reason may be that the ES population is adapted against biotic stresses, including pathogens like fungi and bacteria, while the AS population is adapted mainly against climatic stresses, including solar radiation, heat, and drought.

The obvious difference in Bx production of roots and shoots of the same seedlings that have the same genetic background at the microscale model of ECI and then was grown under a common garden experiment in the Sapir greenhouse could result from different environmental stresses in the soil (roots) and air (shoots) of the greenhouse. The differences between the seedlings of the opposite slopes at ECI are based on genetic differences. The present benzoxazinoid study complements and corroborates the study of resistance of wild emmer wheat to powdery mildew [32]. Fungal genetic resistance to powdery mildew evolved precisely where powdery mildew flourishes, i.e., on the ES rather than on the AS.

At the end of our Hx experiment (6 days after seeding), in ES seedlings we found intermediate concentration (5.2nmol/mg FW) of DIMBOA in the roots with much less DIBOA (2.1nmol/mg FW), while in the same seedlings, there was almost no DIMBOA (0.3nmol/mg FW) in the shoots, but instead an equal intermediate concentration of DIBOA (5.0nmol/mg FW) was determined. The reason for this phenomenon is the different bio-threats from the air to the shoots compared to those that operate in the soil and trigger the production of defense compounds in the roots. A similar phenomenon of these two defenses compounds has been found in rye [40]. In our study, the concentration of the Benzoxazinoids compounds was significantly higher in the roots than in the shoots. The same was found in additional studies that were conducted in greenhouses; however, this was in contrast to a review [40–41] that found the defense compounds had higher concentrations in shoots than in roots. This result may be because they were measured in nature and not in a greenhouse.

Future research could focus on in situ situations at ECI and when different stresses trigger the production of defense metabolites. Likewise, we could add epigenetics in the study of the in-situ populations at ECI, and possibly identify the specific stresses causing the triggering of the defense metabolites.

## Appendix:

**The MATLAB script**:

%% Import the data

    [~, ~, raw] = xlsread('C:\Users\user\Downloads\Yuval Research\衬ລ魠21_7.xlsx','⩬饯1','A3:I14');

    %% Create output variable

    data = reshape([raw{:}],size(raw));

    %% Allocate imported array to column variable names

    ES_Root _DIBOAGlc = data(:,1);

    ES_Root _DIMBOAGlc = data(:,2);

    ES_Shoot _DIBOAGlc = data(:,3);

    ES_Shoot _DIMBOAGlc = data(:,4);

    AS_Root _DIBOAGlc = data(:,5);

    AS_Root _DIMBOAGlc = data(:,6);

    AS_Shoot _DIBOAGlc = data(:,7);

    AS_Shoot _DIMBOAGlc = data(:,8);

    Time = data(:,9);

    %% Clear temporary variables

    clearvars data raw;

    x = Time;

    figure;

    Z = cat(2,AS_Root _DIMBOAGlc,AS_Root _DIBOAGlc,ES_Root _DIMBOAGlc,ES_Root _DIBOAGlc);

    yy = cat(1,' ',' ',' ',' ');

    colormap(jet(216))

    b = bar(x,Z);

    set(gca,'XTickLabel',x);

    set(gca,'FontWeight','bold');

    set(gca,'FontSize',18);

    % set(gca,'YTickLabel',x);

    % title('Root nmol/mg FW values for DIMBOA-Glc & DIBOA-Glc over time');

    title('Root');

    % xlabel('Molecule Name');%,'FontWeight','bold');

  xlabel('Hours after seed (Hr)','FontWeight','bold');

  ylabel('nmol/mg FW','FontWeight','bold');

  % campos ([-10.4880 9.0789 203.2587]);

  colorbar

  % saveas(gcf,'Fig 3A','tiff');

  figure;

  Z = cat(2,AS_Shoot _DIMBOAGlc,AS_Shoot _DIBOAGlc,ES_Shoot _DIMBOAGlc,ES_Shoot _DIBOAGlc);

  yy = cat(1,' ',' ',' ',' ');

  colormap(jet(216))

  b = bar(x,Z);

  set(gca,'XTickLabel',x);

  set(gca,'FontWeight','bold');

  set(gca,'FontSize',18);

  % set(gca,'YTickLabel',x);

  % title('Shoot nmol/mg FW values for DIMBOA-Glc & DIBOA-Glc over time');

  title('Shoot');

% xlabel('Molecule Name');%,'FontWeight','bold');

xlabel('Hours after seed (Hr)','FontWeight','bold');

ylabel('nmol/mg FW','FontWeight','bold');

% campos ([-10.4880 9.0789 203.2587]);

colorbar

% saveas(gcf,'Fig 3B','tiff');
